# Airline capacity distribution under financial budget and resource consideration

**DOI:** 10.1007/s10878-023-01055-0

**Published:** 2023-06-12

**Authors:** Jing Zhou

**Affiliations:** School of Economics and Management, Tongji University, Shanghai, China

**Keywords:** Airline capacity distribution, Financial budget, Fleet introduction, Variable neighborhood search, Branch-and-bound

## Abstract

Capacity distribution is a challenging issue for an airline under financial budget and resource consideration. It is a large-scale optimization problem covering both long-term planning and short-term operating arrangements. This study investigates on the airline capacity distribution problem with financial budget and resource consideration. It contains subproblems of financial budget arrangement, fleet introduction, and fleet assignment. Among them, financial budget is arranged in multiple decision periods, fleet introduction is decided under fixed time points, while fleet assignment is decided under all available time points. To tackle this problem, an integer programming model is formulated for descriptions. Then, an integrated algorithm of modified Variable Neighborhood Search (VNS) and Branch-and-bound (B&B) strategy is developed to find solutions. In detail, a greedy heuristic approach is utilized to generate an initial solution for fleet introduction, the modified B&B strategy is utilized to generate the optimal solution for fleet assignment and the modified VNS is applied to update current solution for a new one with better quality. In addition, budget limit checks are added for financial budget arrangements. Finally, the hybrid algorithm is tested on efficiency and stability. It is also compared to other algorithms which replace the modified VNS by basic VNS, differential evolution and genetic algorithm. Computational results show that performance of our approach is powerful in terms of objective value, convergence speed and stability.

## Introduction

Airline capacity distribution problem (ACDP) is a combination of subproblems which contain financial budget distribution, fleet introduction, and fleet assignment. Airlines usually deal with this problem in batches (Belobaba et al. 2019). For example, United Airlines (2023) introduces its fleets in batches because they are usually ordered and delivered in fixed time points due to a manufacturer’s production costs. Therefore, an airline plans to distribute its long-term financial budget into multiple short terms and then decides on fleet introduction and assignment, which is more complex than an individual fleet introduction and assignment problem. This means that taking financial budget and resource of ACDP into consideration is essential.

In detail, ACDP can be processed as followings. Firstly, a long-term financial budget is distributed into multiple short terms. Secondly, in the beginning of each short term, distributed financial budget is utilized for fleet introduction decisions, containing when to obtain fleets, whether to buy or lease fleets, and which type of fleets to choose. After this process, numbers of different fleet types are determined for fleet assignment. Finally, fleet assignment assigns executed flights with a specific fleet type.

ACDP is a NP-hard optimization problem with high complexity in an airline’s planning and operating process. In recent years, airline network expansion and flight frequency growth make ACDP more complex than before (Development Planning Department of Civil Aviation Administration of China 2023). In addition, unexpected public events can interfere with an airline’s capacity arrangement. For example, travel restrictions during COVID-19 force airlines to rearrange their capacity. The above factors cause improper ACDP arrangements, which then lead to a large proportion of abnormal flights. According to Civil Aviation Administration of China (2023), apart from bad weather and temporary military control, improper ACDP arrangement is the largest cause which accounts for 15.28% of all abnormal flights. In the US, according to the latest flight data from Bureau of Transportation Statistics (2023), the improper ACDP arrangement ranks first among all the reasons for the abnormal flights, which accounts for 30.48% of a total proportion. These numbers indicate that efficiency of airline capacity distribution can be improved considerably to capture more demands and save fleet resources.

As mentioned above, airline capacity is under pressure to deal with the booming industry and public events. Consequently, ACDP is receiving more and more attention. ACDP mainly has three subproblems containing budget distribution, fleet introduction, and fleet assignment. The first subproblem, budget distribution, is a strategic planning arrangement. In this process, an airline generally spends a large proportion of budgets for regular fleet introduction (Assaf 2009). Consequently, an airline tends to distribute long-term budgets into short-term pieces and then introduce fleets in batches (Moon et al. 2015). Oum et al. (2000) furtherly calculate an optimal buy-or-lease proportion on budget distribution, but they have not considered multi-period decisions. The second subproblem, namely fleet introduction, can be constructed in a linear programming model and solved by CPLEX or dynamic programming (Hsu et al. 2011; Bazargan and Hartman 2012). But a limitation for CPLEX and dynamic programming is that they can only deal with small-scale cases, which is far from reality. The third subproblem, fleet assignment, is studied by Abara (1989) and Hane et al. (1995). It is formulated by connection network and time–space network respectively. Then, spilled passengers capture is added to fleet assignment and this more realistic problem can be solved by heuristic method (Barnhart et al. 2009). More recently, fleet assignment has been integrated with other operating procedures in the airline industry containing timetabling design, aircraft routing and crew scheduling (Faust et al. 2017; Kenan et al. 2018; Birolini et al. 2021a). CPLEX and heuristic method are utilized to solve these integrated problems. Although the heuristic method like genetic algorithm can deal with large-scale cases, it is limited to specially-designed settings and cases.

The subproblems of ACDP have been studied individually and integrated with other operating processes. However, they have not been considered as a whole as ACDP, which is a major decision for an airline’s panning and operating processes. By optimizing this problem, an airline can save valuable capacity resources and improve profitability. As this problem has three stages, variable dimension is high, which makes it invalid to utilize enumeration method.

There are several studies on similar problems shown on Table 1 offering insights on algorithm design for our problem. A flowshop problem in manufacture industry is explored with two stages of sorting and assignment by Ewa (2014). A heuristic priority rule and column generation are designed to minimize makespan. A similar two-stage problem in surgery arrangement is studied by Wang et al. (2015). This problem is more complex due to weekly and bi-objective consideration. It is then solved by a discrete particle swarm optimization (PSO) algorithm. Additionally, fixed setup time is taken into consideration and the problem is solved by local search (Li et al. 2018). Then, batch processors and multi-type resources are added to problem settings and genetic algorithm is utilized to search satisfying solutions (Qin et al. 2019; Sun et al. 2020).

**Table 1:** Summary of literature for multi-stage optimization problems

Literature	Industry	Planning interval	Phase	Objective function	Algorithm
Ewa (2014)	Manufacture	Periodically	Sorting + Scheduling	Minimize makespan	A heuristic with priority rules and column generation
Wang et al. (2015)	Healthcare	Weekly	Scheduling + assignment + sorting	Maximize patient satisfaction and minimize hospital operating costs	A discrete PSO algorithm
Li et al. (2018)	Energy	Daily	Assignment + scheduling	Minimize makespan and energy consumptions	A heuristic method based on local search
Qin et al. (2019)	Manufacture	Daily	Assignment + sorting	Minimize makespan	Genetic algorithm
Sun et al. (2020)	Warehouse	Daily	Assignment + sorting	Minimize makespan	Genetic algorithm
Pei et al. (2019)	Manufacture	Periodically	Planning + assignment + scheduling	Minimize maximum completion time of all jobs on each machine	A hybrid algorithm of BA and VNS
Rezgui et al. (2019)	Vehicle	Periodically	Planning + routing	Minimize acquisition, travel and recharging costs of electrical fleets	A VNS algorithm
Zhu et al. (2020)	Healthcare	Weekly	Planning + assignment	Minimize patient waiting and operating room overtime costs	A hybrid algorithm of GWO and VNS
Fan et al. (2020)	Healthcare	Daily	Planning + scheduling	Minimize completion time of all patients in ophthalmology clinic	A hybrid algorithm of EDA and VNS
Zhang et al. (2021)	Manufacture	Periodically	Assignment + sorting	Minimize maximum completion time	A collaborative VND algorithm
Tao et al. (2022)	Manufacture	Daily	Sorting + assignment	Minimize completion time	A self-adaptive artificial bee colony algorithm
Samanta et al. (2022)	Security	Daily	Routing + scheduling	Maximize probability of interdiction	A VNS-based metaheuristic approach
This study	Airline	Periodically	Planning + assignment	Max operating profits minus fleet introduction costs	A hybrid algorithm of modified B&B and VNS

More recently, metaheuristic methods including variable neighborhood search (VNS) are applied. Rezgui et al. (2019) apply VNS to solve an integrated problem of fleet introduction and routing for electrical vehicles. Even a part of VNS, namely variable neighborhood descent (VND) algorithm can be applied to solve a hybrid problem of assignment and sorting problem in manufacture industry with additional consistent sublots (Zhang et al. 2021). VNS can also be integrated with Bat algorithm (BA) by Pei et al. (2019) to solve a serial-batching scheduling problem with resource, budget, setup time and multiple manufacturers considerations. The hybrid VNS-BA algorithm performs better than BA, VNS, and PSO. Similarly, Zhu et al. (2020) develop a hybrid algorithm combining Grey Wolf Optimizer (GWO) with VNS to solve a three-stage dynamic operating room scheduling problem. Fan et al. (2020) replace GWO with estimation of distribution algorithm (EDA) and also combine EDA with VNS for a patient scheduling problem. Then, Samanta et al. (2022) innovatively extend security routing-scheduling problem with single decision maker to double decision makers and build a parallelized framework according to game theory. This large-scale problem is also dealt with a VNS-based metaheuristic approach. Tao et al. (2022) develop a similar metaheuristic method with additional self-adaptive strategy.

According to studies above, VNS is an efficient framework to deal with multi-stage optimization problems which are similar to ACDP. In addition, to improve solution quality, exact method can be incorporated into VNS framework. As a result, in this study, ACDP under financial budget and resource consideration is presented and a hybrid algorithm of VNS and an exact method is proposed to maximize profits. The main contributions are as follows: (1) ACDP under financial budget and resource consideration is described mathematically as an integer programming model; (2) a greedy heuristic approach is designed to improve quality of initial solution; (3) VNS encoding process is utilized to integrate budget distribution and fleet introduction for dimension reduction; (4) a shaking strategy and special neighborhood structure are applied to generate efficient perturbation solutions; (5) an exact method is applied to improve solution quality; (6) the hybrid algorithm is tested on efficiency and stability and compared to other algorithms.

The remaining chapters of this study are arranged below. Section 2 illustrates the description of ACDP. Next, a mathematical model is formulated in Sect. 3. Then, to solve this problem, a modified VNS-B&B algorithm is designed and illustrated in Sect. 4. Section 5 conducts computational experiments for the designed algorithm and compare its performance with other algorithms. Finally, conclusions and future works are described in the last section.

## Notations and problem statement

In this study, ACDP under financial budget and resource consideration is studied. It is an integration problem involving three stages: budget distribution, fleet introduction, and fleet assignment. The notations used throughout this paper are given in Table 2.

**Table 2:** Notations

Notation	Definition
*Sets*	
$F$	Set of fleet types, indexed by *f*
$L$	Set of flight legs scheduled, indexed by *l*
$R$	Set of routes scheduled, indexed by *r*, *r*(*l*) means a route *r* including leg *l*
$H$	Set of time periods, indexed by *h*
$Y$	Set of years in each time period, indexed by *y*
*Parameters*	
$c_{fr}$	Cost of a route *r* with assigned fleet type *f*, $f \in F,r \in R$
$r_{l}$	Ticket revenue for flight leg *l*, $l \in L$
$Cap_{f}$	Capacity of fleet type *f*, $f \in F$
$D_{ly}$	Passenger demand for flight legs *l* in period *h*, $l \in L, \,y \in h,\,h \in H$
$p_{f}$	Price of a bought aircraft with fleet type *f, *$f \in F$
$l_{f}$	Price of a leased aircraft with fleet type *f, *$f \in F$
$b_{h}$	Upper limit of financial budget proportion in period *h*, $l \in L, \,h \in H$
$B$	Total financial budget
*Decision variables*	
$x_{fry}$	A binary variable which equals 1 if the fleet type *f* is assigned to leg *l* in year *y*, $f \in F,\, l \in L,\,y \in h,\,h \in H$
$q_{ly}$	Number of passengers on flight leg *l* in period *h*, $l \in L,\,y \in h,\,h \in H$
$P_{fh}$	Number of bought aircraft with fleet type *f* in stage *h, *$f \in F,\,h \in H$
$L_{fy}$	Number of leased aircraft with fleet type *f* in stage *h, *$f \in F, y \in h,h \in H$

During the first stage, a long planning term is divided as a set of $$H $$ periods. Each period $$h$$ furtherly contains $$Y$$ years, with the beginning of each year $$y$$ as a decision time point. In each period $$h$$, an airline has a financial budget $$b_{h}$$ to decide on numbers of purchased and leased aircraft $$P_{fh} {/}L_{fy}$$ for all fleet types in set $$F$$. Purchasing can only be conducted at the beginning of each period $$h$$, while leasing can be conducted at the beginning of each year $$y$$. Total fleet introduction costs should not exceed total financial budget $$B$$.

**Fig. 1:**
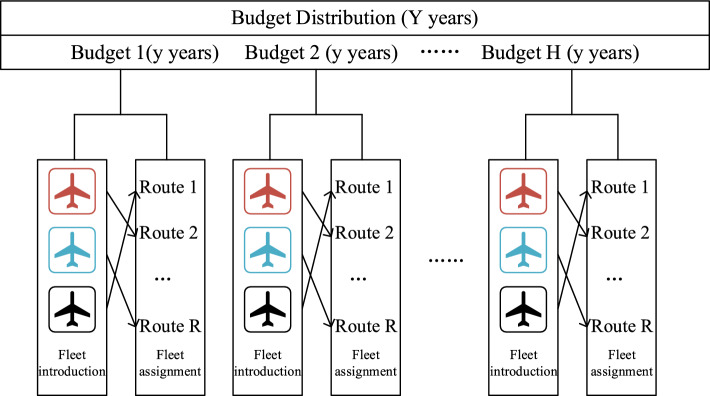
Framework of airline capacity distribution under financial budget and resource consideration

Additionally, a proportion upper limitation $b_{h}$ is set in each period $h$. Thus, total flight numbers of all fleet types can be determined for fleet assignment. At last, in each year $y$ with determined fleet numbers, each flight mission $r$ can be assigned with a suitable fleet $f$ until there is no available fleet left. Each flight mission $r$ contains multiple stops and a flight between two stops is defined as a leg $l$. Each leg $l$ can have a passenger demand $q_{ly}$. This study focuses on an airline’s perspective and assumes that there are no limits on numbers of fleets flowing into and out of all the stops. A framework of the studied problem is shown in Fig. 1.

For example, an airline’s fleet prices, flying missions, flight demands and budget limits are defined in Tables 3, 4 and 5. Because most airlines keep data privacy, direct and complete company-specific data is difficult to obtain. As a result, open websites and reports are referred for numerical settings. To be specific, fleet capacity, fleet prices, total budget volume and period limit numbers, are estimated based on open reports on Statista (Erick 2022a, b). Only three-stop routes with one origin, mid-stop, and destination are considered, which are common choices for an airline. Ticket prices and passenger demands are generated based on an official website-Civil Aviation Administration of China (2022). In detail, from Table 3, purchase prices are set as four times bigger than lease prices. This indicates that, for the same fleet type, it is more profitable to buy a fleet rather than lease if it is utilized for more than 5 years. Table 4 specifies flights in each flying mission. A flight means one departure and one land from one stop to another, each mission can have multiple flights. Table 5 presents budget limit proportions in each planning period and flight demands in each year.

**Table 3:** Fleet prices

Fleet	Name	Seat	Purchase price	Lease price
1	737	150	1 × 10^8^	2 × 10^7^
2	757	200	2 × 10^8^	4 × 10^7^
3	787	300	4 × 10^8^	1 × 10^8^

**Table 4:** Flying missions

Mission	Stop1	Stop2	Stop3	Distance
1	A	B	A	1200
2	A	C	A	800
3	B	C	B	1600

**Table 5:** Budget limits and flight demands in planning periods

Period					H1		H2		H3		H4		H5	
Budget limit proportion (total budget: 8 × 10^8^)					50%		30%		20%		20%		20%	
Flight	Mission	Origin	Destination	Price	Y1	Y2	Y3	Y4	Y5	Y6	Y7	Y8	Y9	Y10
1	1	A	B	300	200	200	200	200	200	200	200	200	0	0
2	1	B	A	300	200	200	200	200	200	200	200	200	0	0
3	2	A	C	200	0	0	0	0	0	0	150	150	150	150
4	2	C	A	200	0	0	0	0	0	0	150	150	150	150
5	3	B	C	400	300	300	300	300	300	300	300	300	300	300
6	3	C	B	400	300	300	300	300	300	300	300	300	300	300

According to the example above, total financial budget is set as 8 × 10^8^. Total planning term is 10 years and divided into 5 periods with 2 years in the same period. Then, the budget can be distributed as 4 × 10^8^, 2 × 10^8^,0, 1 × 10^8^ and 0 from H1–H5, not exceeding the proportion limit in each period of 50%, 30%, 20%, 20% and 20%. Then, the airline decides on fleet introduction. There are three decisions containing buying one 787 in H1, one 757 in H2 and leasing one 737 in H4. These decisions are shown in a square box, a circle and a triangle respectively in Table 6. As a result, in H1, there is one fleet of 787 in each period; in H4, there are three fleets of 737, 757 and 787 in each period; in other periods, there are two fleets of 757 and 787 in each period.

**Table 6:**
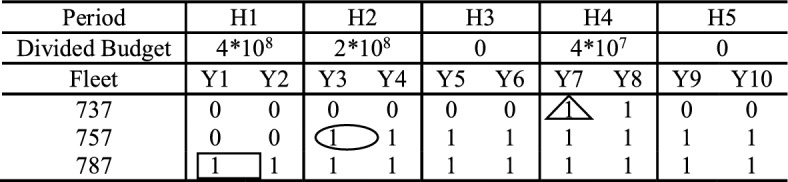
Example of budget distribution and fleet introduction results

After fleet numbers are determined, an airline assigns available fleets to flying missions in all planning years. Results are shown in Table 7. Each flying mission in each year is assigned a fleet according to matching degree of fleet capacity and flying mission demand. Specifically, one 737 is assigned to Mission 2 from Y7 to Y8; one 757 is assigned to Mission 1 from Y3 to Y8 and Mission 2 from Y9 to Y10; one 787 is assigned to Route 3 from H1 to H10.

**Table 7:** Example of fleet assignment results

Period	H1		H2		H3		H4		H5	
Fleet	Y1	Y2	Y3	Y4	Y5	Y6	Y7	Y8	Y9	Y10
737	–	–	–	–	–	–	R2	R2	–	–
757	–	–	R1	R1	R1	R1	R1	R1	R2	R2
787	R3	R3	R3	R3	R3	R3	R3	R3	R3	R3

To summarize, for our ACDP study, a combination of subproblems containing budget distribution, fleet introduction, and fleet assignment, the following decisions should be made:

1. How many budgets to be distributed in each planning period,
2. Which time point is chosen to buy or lease an aircraft,
3. Which type of aircraft is chosen,
4. How many aircrafts is bought or leased,
5. How to match flying missions with suitable available aircrafts.

Among the decisions, (1) belongs to budget distribution, (2–4) belong to fleet introduction and (5) belongs to fleet assignment. In the next sections, this problem is formulated as an integer programming model and then valuable solutions are searched by a designed hybrid algorithm.

## Mathematical modeling

According to descriptions and assumptions above, an integer programming model for ACDP is constructed. As mentioned before, there are mainly three parts of ACDP containing financial budget distribution, fleet introduction, and fleet assignment. The fleet introduction part refers to formulation of Bazargan and Hartman (2012). The fleet assignment part refers to formulation of Xu et al. (2021). Then, to incorporate financial budget distribution, total and period budget limits are added in constraints. The model of ACDP can be described as following:

1 $$ {\text{Maximize}}:\begin{array}{*{20}c} {\mathop \sum \limits_{h \in H} \left( {\mathop \sum \limits_{y \in h} \left( {\mathop \sum \limits_{l \in L} r_{l} q_{ly} - \mathop \sum \limits_{f \in F} \mathop \sum \limits_{r \in R} c_{fr} x_{fry} - \mathop \sum \limits_{f \in F} l_{f} L_{fy} } \right) - \mathop \sum \limits_{f \in F} p_{f} P_{fh} } \right)} \\ \end{array} $$

2 $$ \begin{aligned} & {\text{Subject to}}: \\ & \,\begin{array}{*{20}c} {\mathop \sum \limits_{f \in F} x_{fry} \le 1 \forall r \in R,y \in h, h \in H} \\ \end{array} \\ \end{aligned} $$

3 $$ \begin{array}{*{20}c} {\mathop \sum \limits_{r \in R} x_{fry} \le L_{fy} + P_{fh} + \mathop \sum \limits_{m = 0}^{h - 1} P_{fm} \forall f \in F,y \in h,h \in H} \\ \end{array} $$

4 $$ \begin{array}{*{20}c} {q_{ly} \le D_{ly} \forall l \in L,\,y \in h,\,h \in H} \\ \end{array} $$

5 $$ \begin{array}{*{20}c} {q_{ly} \le \mathop \sum \limits_{f \in F} Cap_{f} x_{fr\left( l \right)y} \forall l \in r\left( l \right),\,r\left( l \right) \in R,\, y \in h,\,h \in H} \\ \end{array} $$

6 $$ \begin{array}{*{20}c} {\mathop \sum \limits_{h \in H} \left( {\mathop \sum \limits_{y \in h} \mathop \sum \limits_{f \in F} l_{f} L_{fy} + \mathop \sum \limits_{f \in F} p_{f} P_{fh} } \right) \le B} \\ \end{array} $$

7 $$ \begin{array}{*{20}c} {\mathop \sum \limits_{y \in h} \mathop \sum \limits_{f \in F} l_{f} L_{fy} + \mathop \sum \limits_{f \in F} p_{f} P_{fh} \le b_{h} \forall h \in H} \\ \end{array} $$

8 $$ \begin{array}{*{20}c} {x_{fry} \in \left\{ {0,1} \right\}, q_{ly} , P_{fh} ,L_{fy} \in N} \\ \end{array} $$

Equation (1) illustrates that the objective is to maximize an airline’s profits. Its value calculated by flying mission revenues minus operating costs and fleet introduction costs. In detail, flying mission revenues are calculated in missions, which indicate all passenger ticket revenues in missions of all executing routes, while operating costs contain costs are calculated in routes. Fleet introduction costs contain purchase and lease costs, calculated in periods and years respectively.

Equation (2) promises that in each fleet assignment decision point, a route can choose no more than one available fleet to execute missions. The decision point is yearly in this study. Then, Eq. (3) connects fleet assignment and introduction. It ensures that in the same point, to sum up all routes, number of occupied fleets cannot exceed upper bound of available ones. The available ones indicate all introduced fleets containing leased fleets in this year, purchased fleets in this period which the year belongs to and all previous periods. Equations (4) and (5) guarantee that realized passenger demand in each flying mission of each route is no more than requested passenger demand and fleet capacity respectively. Equations (6) and (7) indicate a corresponding relationship between budget distribution and fleet introduction. Equation (6) guarantees that total fleet introduction costs cannot exceed total financial budget, while Eq. (7) specifies that in each period, fleet introduction costs cannot exceed periodically upper bounds. Equation (8) defines the nature of all decision variables.

## Proposed algorithm

This section introduces the proposed hybrid algorithm of VNS and B&B in detail. Firstly, algorithm design motivations and framework are illustrated. Secondly, with regards to problem-specific characteristics, algorithm components, involving initial solution generation, encoding, decoding, modified B&B strategy and VND process, are presented respectively. Finally, the whole procedure of the hybrid algorithm for addressing ACDP is summarized.

### Motivations and algorithm framework

In ACDP, there exist mainly three subproblems containing budget distribution, fleet introduction, and fleet assignment. They are considered simultaneously. Since these subproblems are dependent, the whole problem has NP-hard property and its various variables cannot be dealt efficiently with exact methods in limited time. As a result, we adopt a VNS metaheuristic framework to solve this problem. In addition, as one of the subproblems, fleet assignment, can be handled with B&B (Morrison et al. 2016). By integrating a VNS framework and B&B strategy, a hybrid algorithm is designed to give attention to both running speed and solution quality.

**Fig. 2:**
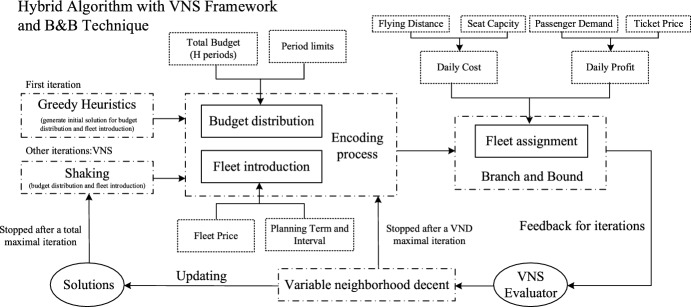
Framework of the integrated problem of fleet introduction and assignment

Figure 2 shows procedures of the algorithm in detail. Firstly, if the initial solution is generated randomly, our algorithm is easy to stuck in a local optimum and difficult to find a satisfying finial result in limited time. Thus, greedy heuristics based on our problem are applied to generate various solutions for budget distribution and fleet introduction. Then, one of them with the best performance on objective function (1) is picked as the initial solution to start the algorithm. Secondly, two subproblems, budget distribution and fleet introduction can be cooperated and encoded. For the last subproblem, fleet assignment, B&B strategy is utilized to provide results. Solutions of these three subproblems can generate a VNS evaluator, which equals to the objective function (1) in Sect. 3. Thirdly, based on the current solutions, VND with specific neighborhood design is conducted to search for better solutions. Finally, after solution updating, a shaking operation is adopted to start a new iteration until the maximal one is ended.

### Initial solution generation

**Fig. 3:**
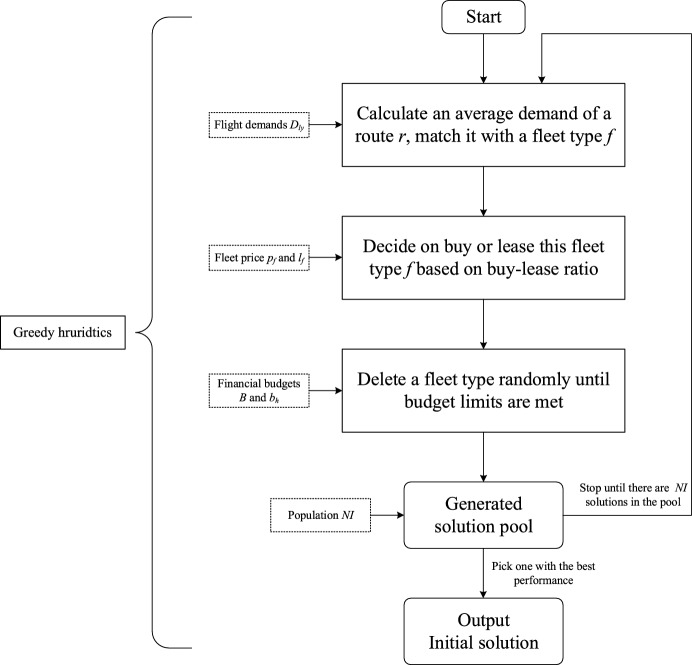
Framework of greedy heuristics

To start the hybrid algorithm, an initial solution is generated by greedy heuristics shown in Fig. 3 based on problem-specific characteristics. To be specific, passenger demand $D_{ly}$ (predefined) domains profits in objective function (1). It can be utilized to conduct greedy strategy. Firstly, for fleet assignment, assuming that a fleet can only execute one route $r$ and there is no transformation between routes, a fleet with the nearest capacity to a route’s average passenger demand is assigned to the same route. Secondly, once a fleet type $f$ is determined, buying or leasing is decided by the fleet type’s buy-lease ratio. For example, in Table 3, if a fleet 737 is executed for more than 5 years, buying it is less costly than leasing it. Thirdly, total and period budget limits are added. If fleet introduction costs exceed a period $H$ budget limit, a fleet bought or leased in the same period is deleted randomly until the budget limit is met. For total budget limit, fleets in all periods can be deleted randomly until the budget limit is met. After that, because fleet introduction decisions are changed, fleet assignment, which is correlated, should be updated with B&B strategy to calculate objective function value. Finally, due to randomness in budget distribution process, a population of solutions is generated iteratively and one with the best performance on objective value is chosen as the initial solution.

**Fig. 4:**
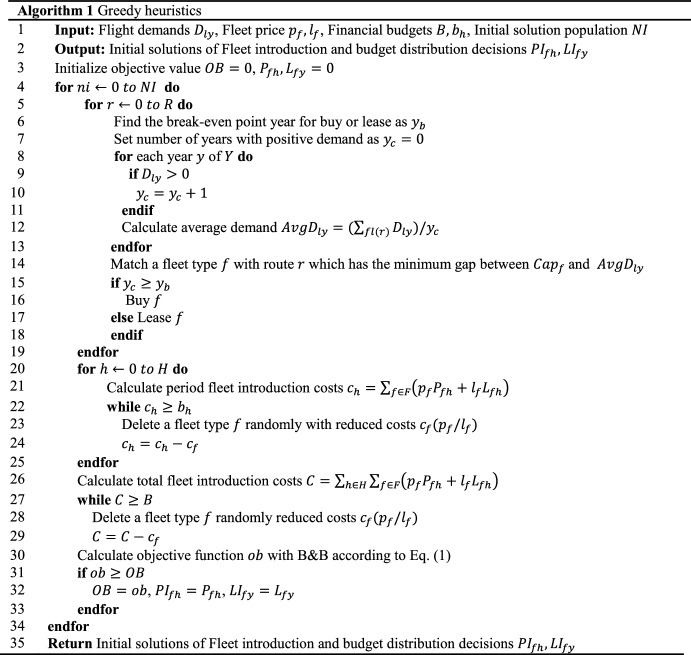
Initial solution generation algorithm

Pseudocode of the initial solution generation process is illustrated in Fig. 4. Flight demands $D_{ly}$, financial budgets $B, b_{h}$, initial solution population $NI$ are imported into the greedy heuristics and initial solutions of fleet introduction and budget distribution decisions $PI_{fh} {/}LI_{fy}$ are returned for a subsequent encoding process.

### VNS encoding and decoding process

**Fig. 5:**
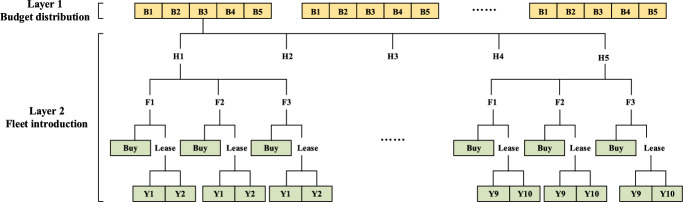
Joint encoding process

Encoding process plays an important role in the VNS framework. It needs to construct a suitable solution structure for consecutive search efficiency. As described above, three subproblems of ACDP involving budget distribution, fleet introduction, and fleet assignment should be considered. For fleet assignment, due to discussions in 4.1, B&B strategy can be applied and its details is illustrated in Sect. 4.4 illustrates. For budget distribution and fleet introduction, if they are encoded jointly, both spent budget in all periods $B_{h}$ and bought/leased numbers of all fleet types in all periods $P_{fh} {/}L_{fy}$ should be set as variables illustrated in Fig. 5. This encoding has two major layers containing budget distribution and fleet introduction. For the latter layer, it even has three sub-layers, which leads to great difficulty in subsequent search steps.

**Fig. 6:**
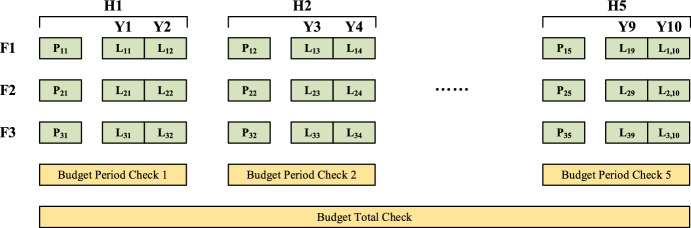
Single encoding process with budget checks

To improve the encoding design, fleet introduction is considered singly to reduce variable dimensions illustrated in Fig. 6. To be specific, the three sub-layers of fleet introduction variables can be linearized by period, fleet type and buy-or-lease choices. Budget distribution considerations are transferred to budget limit checks both totally and periodically which can be programmed in the following search steps. Thus, a total budget $B_{h}$ and its periodical arrangement $b_{h}$ can be deleted from our formulation and algorithm framework. This operation reduces not only orders of variables in encoding process, but also possibility of illegal solution generations.

The encoding process is prepared for the following VND and shaking operations after solving fleet assignment with B&B strategy. Differently, the decoding process is prepared for fleet assignment in the next step directly. Values of numbers of newly introduced fleets ($P_{fh} ,L_{fy}$) can be extracted from arrays in Fig. 6 with indexes ($H \times \left( {1 + Y} \right)$, $H \times \left( {1 + Y} \right) + 1\sim H \times \left( {1 + Y} \right) + Y$). Based on these values, numbers of all available fleets in different periods and specific years can be calculated as the right hand of Eq. (3). Then, fleet assignment is ready to be solved in the next section.

### Modified B&B strategy

After the encoding process, bought/leased numbers of all fleet types in all periods $P_{fh} {/}L_{fy}$ can be determined. According to our formulation, the third and fourth terms in objective function (1) can be calculated out with constraints (6) and (7) satisfied. To calculate the first and second terms of objective function (1) with all the other constraints satisfied, namely to solve the fleet assignment problem, B&B strategy can be applied. In this study, as mentioned above, the first and second terms of objective function (1) is set as the B&B evaluator and also illustrated in Eq. (9). This evaluator represents an airline’s net operating profits of fleet assignment.

9 $$ \begin{array}{*{20}c} {net\,\,operaitng \,\,profits = \mathop \sum \limits_{l \in L} r_{l} q_{ly} - \mathop \sum \limits_{f \in F} \mathop \sum \limits_{r \in R} c_{fr} x_{fry} } \\ \end{array} $$

Specifically, in Eq. (9), a unit profit $r_{l}$ of a flight leg $l$ (a route $r$ contains several legs $l$) is predefined. Then, realized demand $q_{ly}$ is the minimum value of requested passenger demand $D_{ly}$ and assigned fleet type capacity $Cap_{f}$ shown in Eq. (10). For operating costs, they are calculated in a route unit as Birolini et al. (2021b) estimate in Eq. (11). In Eq. (11), a route’s distance $S_{r}$ is also predefined. In addition, for aircraft maintenance consideration, maintenance can be divided into two types containing route and scheduled maintenance (Zhu 2009). The former is closely related to the flying mission $r$ in this study. Its costs are considered in Eq. (11), which is also a route-based estimation (Birolini et al. 2021b). The latter has a relatively long interval and is usually ignored in estimation.

10 $$ \begin{array}{*{20}c} {q_{ly} = min\left\{ {D_{ly} , Cap_{f} } \right\}} \\ \end{array} $$

11 $$ \begin{array}{*{20}c} {c_{fr} = \left( {S_{r} + 722.0} \right) \times \left( {Cap_{f} + 104.0} \right) \times 0.019} \\ \end{array} $$

**Fig. 7:**
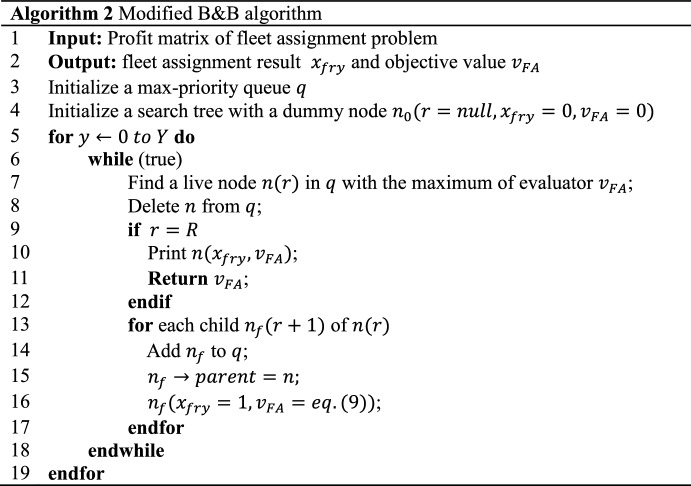
Modified B&B algorithm

Then, there are three rules in B&B involving searching, branching, and bounding which can be modified to adapt to our problem. Firstly, a cyclic best-first search is adopted to find results. A priority queue is built to move the current best solution at the top until the bottom layer is searched. The queue ensures both solution quality and running speed (Morrison et al. 2016). Secondly, in branching process, wide branching is chosen. This mean that one route is set as one layer and different fleet type choices connect two adjacent layers. Compared to binary branching, which determining a yes-or-no problem for all fleet types one by one for each route, wide branching improves search efficiency. Thirdly, if the value of operating profits in Eq. (9) is the lowest among all potential results, this value and its correlated solution should be deleted. The modified B&B strategy is presented in Fig. 7.

### VNS evaluator and neighborhood design

Based on the steps above, both an initial solution and a related objective value of ACDP is prepared. Specifically, greedy heuristics in Sect. 4.2 offer initial solutions of budget distribution and fleet introduction. In addition, the third and fourth terms of objection function Eq. (1) are calculated. Then, encoding results are generated in Sect. 4.3 to be utilized in neighborhood design. Finally, a modified B&B strategy in Sect. 4.4 provides fleet assignment solutions and calculates out the first and second terms of objection function Eq. (1). In this way, VNS evaluator [namely the value of objective function Eq. (1)] and the initial solution of ACDP are ready.

To improve this solution, VND and shaking operations should be applied. In the two operations, neighborhood structures should be designed carefully, because they make considerable effects on solution quality and running speed. Different from a traditional VNS structure, our encoding results have multiple layers involving time periods, buy-or-lease choices and fleet types. As a result, neighborhood structures are designed as followings.

**Fig. 8:**
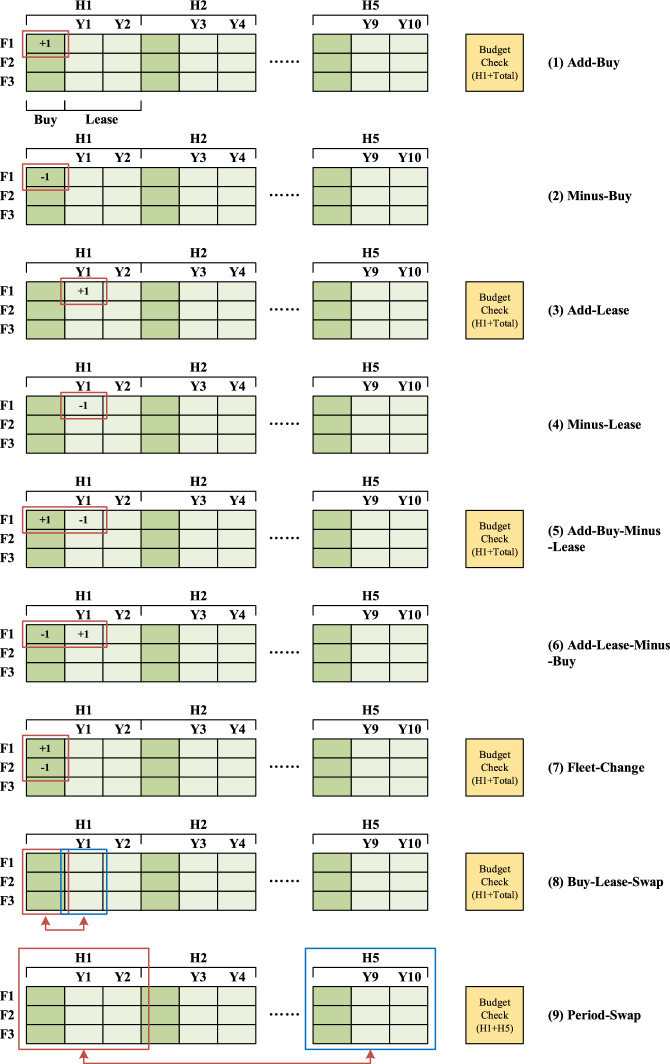
Neighborhood structures with crossover and variation operations

There are nine neighborhood structures to deal with encoding results involving: Add-Buy, Minus-Buy, Add-Lease, Minus-Lease, Add-Buy-Minus-Lease, Add-Lease-Minus-Buy, Fleet-Change, Buy-Lease-Swap, and Period-Swap. They are indexed in Fig. 8 from (1) to (9):

1. Add-Buy: choose a value of bought fleet $P_{fh}$ randomly and add $1$;
2. Minus-Buy: choose a value of bought fleet $P_{fh}$ randomly and minus $1$;
3. Add-Lease: choose a value of leased fleet $L_{fy}$ randomly and add $1$;
4. Minus-Lease: choose a value of leased fleet $L_{fy}$ randomly and minus $1$;
5. Add-Buy-Minus-Lease: combination of (1) and (4);
6. Buy-down-lease-up: combination of (2) and (3);
7. Fleet-Change: change one fleet type to another randomly;
8. Buy-Lease-Swap: swaps values of bought and leased fleets in one period;
9. Yearly swap: choose two years randomly, swap all the fleet numbers.

For these nine structures, (1–4) only change one value in the encoded results, (5–7) change two values, (8) changes six values, while (9) involves two periods and change 18 numbers totally. From a multi-layer view, (1–6) and (8) are concerned with buy-or-lease choices, (7) is related to fleet type decisions, while (9) represents time period choices.

### Budget limit checks

Once a neighborhood of fleet introduction is generated in Fig. 8, a new value of fleet introduction costs replaces the previous one, which is possibly conflicted with constraints (6) and (7). Consequently, budget limits should be considered totally and periodically.

Our budget limit checks are incorporated into neighborhood generation process in Sect. 4.5. When a new code is generated, fleet introduction cost is calculated out according to the third and fourth terms of Eq. (1). Then, it is compared with relative period budgets and total budget (both predefined). For example, for a new code from neighborhood (8), it has changes in period 1, thus, both total budget and budget of this period should be checked, while for a new code from neighborhood (9), it has changes in period 1 and 5, thus, budget of these two periods should be checked. As numbers in this code is swapped between periods, there is no need to check total budget.

After comparison, if the fleet introduction cost of the new code exceeds relative period budgets or total budget, the code is invalid and should be deleted. The previous code is output for VND process.

**Fig. 9:**
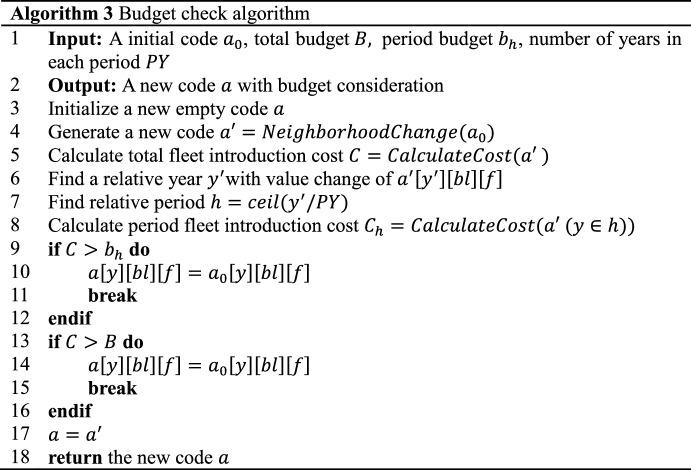
Period and total budget check operations

Algorithms of period and total budget checks are illustrated in Fig. 9. As shown before, an initial code $a_{0}$ is firstly input with three dimensions involving year $y$, buy-or-lease choice $bl$ and fleet type $f$. Secondly, after the neighborhood change as shown in Fig. 8, a new code $a^{\prime}$ is generated and a relative year $y^{\prime}$ with value change of $a^{\prime}\left[ {y^{\prime}} \right]\left[ {bl} \right]\left[ f \right]$ is picked up. Thirdly, a time period $h$ which $y^{\prime}$ belongs to can be found. Then, both total and period ($h$) fleet introduction costs are calculated. They are compared to total and period ($h$) budgets respectively. If the costs exceed budgets, the new code is deleted and the initial one is reused for the following VND process.

## Computational experimentation

This section intends to systematically evaluate our integer programming model and modified VNS-B&B hybrid algorithm. It is compared with three metaheuristics containing basic VNS, differential evolution (DE), and genetic algorithm (GA). They only replace the modified VNS part and incorporate with B&B. Three different sets of benchmarks are set and collected to compare their performance involving objective value, running speed and robustness. All experiments of the modified VNS-B&B hybrid algorithm and compared metaheuristics are coded in C++. They are run on a windows 10 system with a 1.10 GHz Intel Core i7-10710U CPU processor and a 16G RAM.

### Test data

**Fig. 10:**
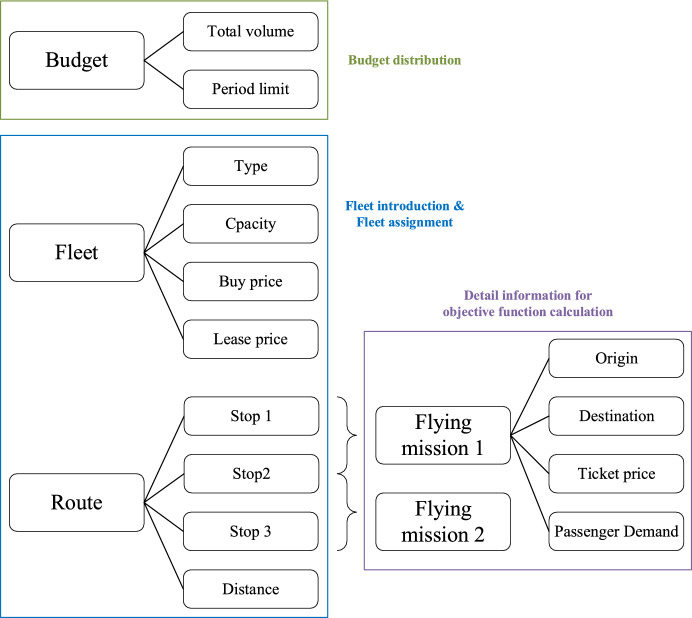
Parameter input structure

To solve ACDP problem, we set four instances scaled with 3, 10, 20, and 50 routes, which covers airlines of small and medium sizes. For each instance, there are four sets of input data involving budget, fleet, route, and flying mission information shown in Fig. 10. Firstly, budget numbers are prepared for budget limits distribution. Secondly, fleet and route settings are predefined for fleet introduction and assignment decisions. Thirdly, flying mission inputs offer details for routes and help objective function calculation.

Data sources are open websites and reports. Because most of airlines keep data privacy, direct and complete company-specific data is difficult to obtain. As a result, open resources are referred for numerical settings. To be specific, total budget volume, period limit numbers, fleet capacity and fleet prices are estimated based on open reports on Statista (Erick 2022a, b). Only three-stop routes are considered in our experiments, which are common choices and consistent to the short-distance calculation in Eq. (11). Ticket prices and passenger demands are generated based on an official website-Civil Aviation Administration of China (2022). The smallest case is illustrated above in Tables 3, 4, and 5 with a scale of 3 routes.

### Parameter settings

To test our modified VNS-B&B algorithm, the modified VNS part is tested and then replaced by other efficient metaheuristics involving DE, GA, and basic VNS. There are various parameters to be predefined before the experiments described in Table 8.

**Table 8:** Parameter settings

Notation	Definition	
$H$	Number of time periods	5
$PY$	Number of years in each time period	2
$Day$	Number of days in each year	365
$R$	Number of routes	3, 10, 20, and 50
$F$	Number of fleet types	3 (737, 757, and 787)
$EX$	Experimental times for each algorithm	20
*VNS*		
$S$	Maximal iterations of VNS (shaking times)	300
$SP$	Population of each shaking operation	100
$L$	Number of VND neighborhoods	9
$LL$	Maximal iterations in each VND neighborhood	10
*Basic VNS*		
$BS$	Maximal iterations of VNS (shaking times)	300
$BSP$	Population of each shaking operation	100
$BL$	Number of VND neighborhoods	4
$BLL$	Maximal iterations in each VND neighborhood	30
*GA*		
$G$	Maximal iterations of GA	300
$P$	Population size	250
$PC$	Probability of crossover	0.9
$PM$	Probability of mutation	0.4
*DE*		
$D$	Maximal iterations of DE	300
$FF$	DE-scaling factor	3
$N$	Population	300
$CR$	Recombination rate of DE	0.8

For all experiments, ACDP is planned in the future 10 years and they are divided into 5 periods with 2 years in each period. Assuming there are 365 days in each year, flying missions are repeated in each day, once a mission is assigned a fleet type, it would repeat in each day. Then, yearly operating profits can be calculated as daily profits multiplied by 365 days, which is a widely used simplification (Kenan et al. 2018). All the four algorithms are conducted for 20 times and in each time the maximal iterations are 300. Then, specific parameters for the modified VNS-B&B algorithm and the other three compared algorithms are also set in Table 8.

For our modified VNS, number of VND neighborhoods $L$ is set as 9 and maximal iterations in each VND neighborhood $LL$ is set as 10. Those numbers in basic VNS are set as 4 and 30 based on Xu et al. (2021). To improve performances, a population of 100 is additionally set in shaking operation in both algorithms ($SP, BSP$). For GA, based on Tang et al. (2021), population size $P$ is set as 250, probabilities of crossover and mutation are set as 0.9 and 0.4 respectively. For DE, according to Kusoncum et al. (2022), population size $N$ is the biggest of all the four algorithms with the number of 300. DE-scaling factor $FF$ and recombination rate $CR$ are set as 3 and 0.8.

### Result analysis

This subsection intends to evaluate the performance of modified VNS-B&B algorithm and compare it with DE-B&B, GA-B&B and basic VNS-B&B algorithms. The first phases of the four hybrid algorithms deal with two subproblems of ACDP containing budget distribution and fleet introduction with metaheuristics. The second phases apply modified B&B strategy to solve fleet assignment, another subproblem of ACDP. The four algorithms are compared on running speeds, objective values and robustness. For robustness, an evaluator named Relative Percent Deviation ($$RPD$$) is applied. All the four algorithms should be conducted for 20 times, thus, both average value $$Avg$$ and best objective value $$Best $$ are prepared. RPD is calculated as deviation between $$Best$$ and $$Avg$$ divided by $$Best$$ and multiplied by 100. The equation of RPD is presented in Eq. (12). A smaller RPD means a smaller deviation between best and average solutions, which indicates a more stable algorithm.

12 $$ \begin{array}{*{20}c} {RPD = \frac{Best - Avg}{{Best}} \times 100} \\ \end{array} $$

Experiments of the four instances in Sect. 5.1 are coded in C++ and run on a windows 10 system with a 1.10 GHz CPU processor and a 16G RAM as mentioned before. After experiments, performances of all the four algorithms are illustrated in Table 9. The first and second column display case indexes and scales. The third column lists the four algorithms to compare their results. Then, the last three columns show three indicators of algorithm performance, containing objective value, RPD and running time.

**Table 9:** Performances for modified VNS/Basic VNS/GA/DE-B&B algorithms

Case	Scale	Algorithm	Objective value	RPD	Running time(s)
1	3 routes	Modified VNS-B&B	*5.82E + 08	*0.00	*4.79
		Basic VNS-B&B	5.53E + 08	2.70	6.46
		GA-B&B	5.78E + 08	0.69	8.25
		DE-B&B	5.48E + 08	5.86	9.46
2	10 routes	Modified VNS-B&B	*1.61E + 09	0.68	*18.60
		Basic VNS-B&B	1.54E + 09	*0.16	28.02
		GA-B&B	1.48E + 09	1.82	45.86
		DE-B&B	1.46E + 09	9.70	52.15
3	20 routes	Modified VNS-B&B	*2.59E + 09	2.81	*49.78
		Basic VNS-B&B	2.31E + 09	*0.54	70.53
		GA-B&B	2.22E + 09	3.08	125.38
		DE-B&B	2.04E + 09	0.59	121.65
4	50 routes	Modified VNS-B&B	*7.00E + 09	*1.67	*265.82
		Basic VNS-B&B	6.28E + 09	2.19	313.50
		GA-B&B	6.21E + 09	2.37	443.83
		DE-B&B	6.94E + 09	3.79	608.59

*represents that an algorithm has the best performance among all algorithms on an indicator in a case

In terms of objective value, the modified VNS-B&B is dominant among all the four algorithms. Because the objective in Eq. (1) is to maximizer total profits, if the value in the fourth column is bigger, the performance of an algorithm is better. In all cases, the modified VNS-B&B algorithm obtains the biggest values of objective with the numbers of 5.82E + 08, 1.61E + 09, 2.59E + 09 and 7.00E + 09. This indicate that the modified VNS-B&B provides the best objective value of all the four algorithms.

In terms of running speed, the modified VNS-B&B also has advantage over the other three algorithms. The running speed is measured as the running time in second, which starts from the beginning of our algorithms and ends after the final iteration is finished. A smaller value of seconds means a better running speed of an algorithm. In all cases, the modified VNS-B&B algorithm obtains the smallest values of running time with the numbers of 4.79 s, 18.60 s, 49.78 s and 265.82 s. To compare it with other algorithms, our modified VNS-B&B is approximately 1/3 faster than basic VNS-B&B and 1/2 faster than GA/DE-B&B. For example, in case 1, the modified VNS-B&B has a running time of 4.79 s while the basic VNS-B&B has a running time of 6.46 s, which means that the former is 25.81% faster than the latter. The proportion for case 2 is 33.62% and similar in other cases.

Comprehensively, although there are adjacent objective values compared to those of modified VNS-B&B, their speeds are considerably slower than those of modified VNS-B&B. For example, the objective value of GA-B&B in case 1 with the number of 5.78E + 08 has the smallest deviation to that of the modified VNS-B&B, while the running time of the former with the number of 4.79 s is considerably slower than that of the latter with the number of 8.25 s. For the objective value of DE-B&B in case 4 with the number of 6.94E + 09, the situation is similar.

In terms of robustness, as mentioned before, RPDs are utilized for evaluations. In Table 9, the modified VNS-B&B algorithm has a relatively good performance on RPDs. Specifically, in case 1 and 4, the RPDs of modified VNS-B&B algorithm are the smallest compared to those of the other three algorithms with values of 0.00 and 1.67 respectively. This indicates that it is the most robust method to solve the ACDP among all four algorithms. In case 2 and 3, although the RPDs of basic VNS-B&B algorithm are the smallest, which means that this algorithm can robustly find similar solutions in 20 repeated experiments, its objective values in Table 9 are considerably smaller than those of modified VNS-B&B, cancelling the robustness advantage of basic VNS-B&B. In addition, the RPDs of modified VNS-B&B algorithm in case 2 and 3 are close to the basic VNS-B&B one with values of 0.68 and 2.81 respectively, which are acceptable.

**Fig. 11:**
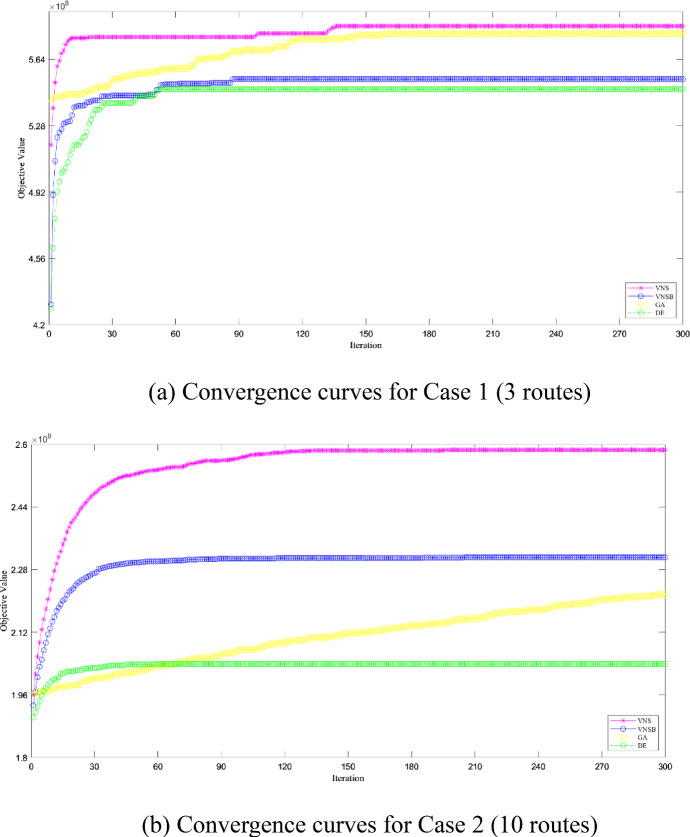

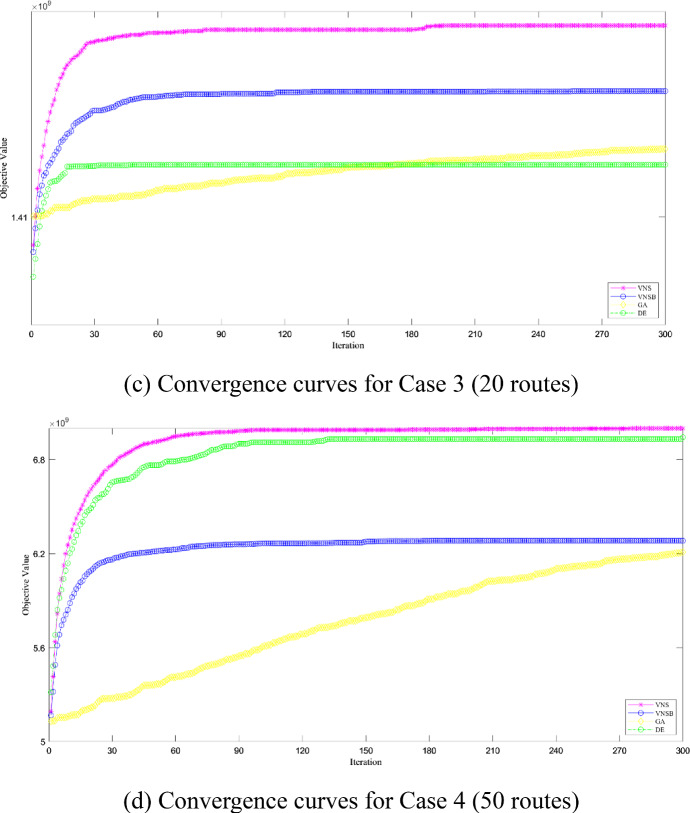
Convergence for Modified VNS/Basic VNS/GA/DE-B&B Algorithm

In detail, in all cases and algorithms, iterative processes are tracked and recorded as convergence curves shown in Fig. 11. For basic VNS and DE-B&B, they converge quickly and both lines are under the line of modified VNS-B&B. For modified VNS and GA-B&B, they both converge slowly but diverge in objective values. Specifically, the line of modified VNS-B&B is on top of GA-B&B and is potential to increase after 300 iterations are over.

**Fig. 12:**
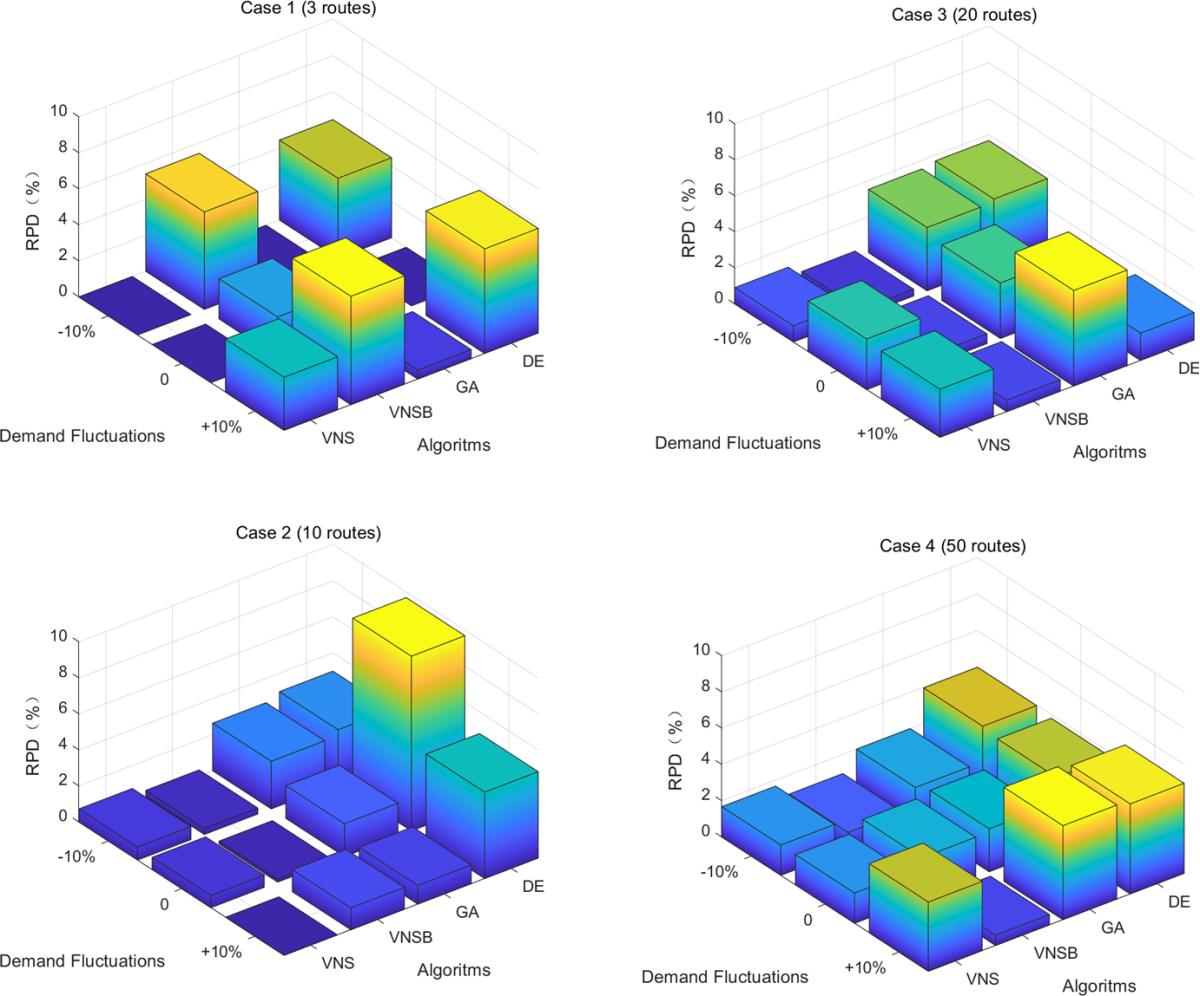
RPDs for Modified VNS/Basic VNS/GA/DE-B&B Algorithm

Furtherly, to test out whether the robustness of modified VNS-B&B algorithm is sensitive to input disturbance, we make changes on input numbers of total budget and passenger demands, which are likely to fluctuate under volatile market conditions. To be specific, total budget and passenger demands are assumed to be 10% more or less than before while the other input numbers remain the same. Then, two more groups of experiments are conduced besides those shown in Table 9. For all the cases, three-dimension graphs on RPDs are illustrated in Fig. 12.

In Fig. 12, for case 1 and 4, although GA-B&B algorithm performs better than modified VNS-B&B algorithm in terms of RPDs, their gaps are small with a largest deviation of approximately 2. Similarly, for case 2 and 3, basic VNS-B&B algorithm performs slightly better than modified VNS-B&B algorithm with a largest deviation of approximately 1. In the other conditions, modified VNS-B&B algorithm domains in terms of RPDs.

To summarize, compared to DE-B&B, GA-B&B, and basic VNS-B&B hybrid algorithms, the modified VNS-B&B algorithm has advantages on objective value and running speed. In terms of robustness, the modified VNS-B&B algorithm performs good and only has slight deviations in limited conditions.

## Conclusion

This paper investigates on the ACDP problem with resource and budget consideration. It is an integration of subproblems containing budget distribution, fleet introduction, and fleet assignment. An integer programming model is formulated to describe the problem and a hybrid algorithm of modified VNS and B&B strategy is designed to find solutions. The hybrid algorithm consists of seven components containing initial solution generation, encoding, decoding, modified B&B, VND and shaking process processes. Moreover, budget limit checks are added once a new neighborhood is generated to avoid invalid solutions and improve search efficiency. The modified VNS-B&B algorithm is compared with the DE/GA/basic VNS-B&B algorithms on objective values, running speeds, and robustness. Experimental results show that the efficiency and stability of the modified VNS-B&B algorithm take advantage over the other compared algorithms.

Our study contributes to reduce dimensions of the complex integrated ACDP problem and provide practical advice for airline planning decisions under budget and resource limits. In the future, relative research can focus on incorporate more limited considerations into ACDP problem and solve it with a more efficient method in larger cases.

## Data Availability

Enquiries about data availability should be directed to the author.

## References

[CR1] Abara J (1989). Applying integer linear programming to the fleet assignment problem. Interfaces.

[CR2] Assaf A (2009). Are U.S. airlines really in crisis?. Tour Manag.

[CR3] Barnhart C, Farahat A, Lohatepanont M (2009). Airline fleet assignment with enhanced revenue modeling. Oper Res.

[CR4] Bazargan M, Hartman J (2012). Aircraft replacement strategy: model and analysis. J Air Transp Manag.

[CR5] Belobaba P, Odoni A, Barnhart C (2019). The global airline industry.

[CR6] Birolini S, Antunes A, Cattaneo M, Malighettia P, Paleari S (2021). Integrated flight scheduling and fleet assignment with improved supply-demand interactions. Transp Res Part b Methodol.

[CR7] Birolini S, Jacquillat A, Cattaneo M, Antunes A (2021). Airline network planning: mixed-integer non-convex optimization with demand-supply interactions. Transp Res Part b Methodol.

[CR8] Bureau of Transportation Statistics (2023) Understanding reporting causes flight delays and cancellations. Available at: https://www.bts.gov/topics/airlines-and-airports/understanding-reporting-causes-flight-delays-and-cancellations. Accessed 12 Apr 2023

[CR9] Civil Aviation Administration of China (2022) Statistics of main production indicators of CAAC in August 2022. Available at: http://www.caac.gov.cn/XXGK/XXGK/TJSJ/202209/P020220920312815593554.pdf. Accessed 8 Jan 2023

[CR10] Civil Aviation Administration of China (2023) Annual report of China's civil aviation industry 2021. Available at: http://www.caac.gov.cn/XXGK/XXGK/TJSJ/202205/P020220518569126412044.pdf. Accessed 9 Apr 2023

[CR11] Development Planning Department of Civil Aviation Administration of China (2023). Learn China’s civil aviation from a statistical perspective 2021.

[CR12] Erick S (2022a) Average new aircraft lease rates worldwide in 2021, by aircraft model. Available at: https://www.statista.com/statistics/1258900/aircraft-lease-rates-aircraft-model. Accessed 8 Jan 2023

[CR13] Erick S (2022b) Average prices for Boeing aircraft as of March 2022b, by Type. Available at: https://www.statista.com/statistics/273941/prices-of-boeing-aircraft-by-type. Accessed 8 Jan 2023

[CR14] Ewa F (2014). A heuristic for scheduling in a two-stage hybrid flowshop with renewable resources shared among the stages. Eur J Oper Res.

[CR15] Fan W, Wang Y, Liu T, Tong G (2020). A patient flow scheduling problem in ophthalmology clinic solved by the hybrid EDA-VNS algorithm. J Comb Optim.

[CR16] Faust O, Gonsch J, Klein R (2017). Demand-oriented integrated scheduling for point-to-point Airlines. Transp Sci.

[CR17] Hane C, Barnhart C, Johnson E, Marsten R, Nemhauser G, Sigismondi G (1995). The fleet assignment problem: solving a large-Scale integer program. Math Program.

[CR501] Hsu C, Li H, Liu S, Chao C (2011). Aircraft replacement scheduling: a dynamic programming approach. Transp Res ETransp Res E.

[CR18] Kenan N, Jebali A, Diabat A (2018). An integrated flight scheduling and fleet assignment problem under uncertainty. Comput Oper Res.

[CR19] Kusoncum C, Sethanan K, Hartl R, Jamrus T (2022). Modified differential evolution and heuristic algorithms for dump tippler machine allocation in a typical sugar mill in Thailand. Oper Res Int Journal.

[CR20] Li J, Sang H, Han Y, Wang C, Gao L (2018). Efficient multi-objective optimization algorithm for hybrid flow shop scheduling problems with setup energy consumptions. J Clean Prod.

[CR21] Moon J, Lee WS, Dattilo J (2015). Determinants of the payout decision in the airline industry. J Air Transp Manag.

[CR22] Morrison D, Jacobson S, Sauppe J, Sewell E (2016). Branch-and-bound algorithms: a survey of recent advances in searching, branching and pruning. Discret Optim.

[CR23] Oum T, Zhang A, Zhang Y (2000). Optimal demand for operating lease of aircraft. Transp Res Part b Methodol.

[CR24] Pei J, Liu X, Fan W, Pardalos PM, Lu S (2019). A hybrid BA-VNS algorithm for coordinated serial-batching scheduling with deteriorating jobs, financial budget, and resource constraint in multiple manufacturers. Omega.

[CR25] Qin M, Wang R, Shi Z, Liu L, Shi L (2019). A genetic programming-based scheduling approach for hybrid flow shop with a batch processor and waiting time constraint. IEEE Trans Autom Sci Eng.

[CR26] Rezgui D, Siala JC, Aggoune-Mtalaa W, Bouziri H (2019). Application of a variable neighborhood search algorithm to a fleet size and mix vehicle routing problem with electric modular vehicles. Comput Ind Eng.

[CR27] Samanta S, Mohandass T, Sen G, Ghosh SK (2022). A VNS-based metaheuristic approach for escape interdiction on Transportation Networks. Comput Ind Eng.

[CR28] Sun B, Zhang X, Qiao H, Li G, Chen Y (2020). Multi-type resources collaborative scheduling in automated warehouse with fuzzy processing time. J Intell Fuzzy Syst.

[CR29] Tang L, D'Ariano A, Xu X, Li Y, Ding X, Samà M (2021). Scheduling local and express trains in suburban rail transit lines: Mixed-integer nonlinear programming and adaptive genetic algorithm. Comput Oper Res.

[CR30] Tao X, Pan Q, Gao L (2022). An efficient self-adaptive artificial bee colony algorithm for the distributed resource-constrained hybrid flowshop problem. Comput Ind Eng.

[CR31] United Airlines (2023) Taking our fleet to new heights. Available at: https://www.united.com/ual/en/us/fly/company/new-and-improved-united-airlines-fleet.html. Accessed 7 Jan 2023

[CR32] Wang Y, Tang J, Pan Z, Yan C (2015). Particle swarm optimization-based planning and scheduling for a laminar-flow operating room with downstream resources. Soft Comput.

[CR33] Xu Y, Wandelt S, Sun X (2021). Airline integrated robust scheduling with a variable neighborhood search-based heuristic. Transp Res Part b Methodol.

[CR34] Zhang B, Pan Q, Meng L (2021). A collaborative variable neighborhood descent algorithm for the hybrid flowshop scheduling problem with consistent sublots. Appl Soft Comput.

[CR35] Zhu J (2009). Air transportation planning.

[CR36] Zhu S, Fan W, Liu T, Tang S, Pardalos PM (2020). Dynamic three-stage operating room scheduling considering patient waiting time and surgical overtime costs. J Comb Optim.

